# An Uncommon Diagnosis of Necrotizing Mastoiditis Presenting as Bell’s Palsy: A Case Report

**DOI:** 10.5811/cpcem.1263

**Published:** 2023-11-08

**Authors:** Parker Maddox, Claire Abramoff

**Affiliations:** * Sidney Kimmel Medical College at Thomas Jefferson University, Philadelphia, Pennsylvania; † Department of Emergency Medicine, Einstein Medical Center Philadelphia, Philadelphia, Pennsylvania

**Keywords:** case report, Bell’s palsy, necrotizing mastoiditis

## Abstract

**Introduction:**

The benign nature of Bell’s palsy has led to a lack of a standardized work-up, and dangerous underlying mimics are at risk of being missed.

**Case Report:**

An 84-year-old female with a history of vertigo presented to the emergency department with a left-sided facial droop consistent with Bell’s palsy. After further work-up, the patient was diagnosed with bilateral necrotizing mastoiditis.

**Conclusion:**

Unilateral facial weakness involving the forehead and palpebral fissures is often diagnosed as idiopathic Bell’s palsy. Various pathologies can present with unilateral facial weakness, and the differential needs to remain broad.

CPC-EM Capsule What do we already know about this clinical entity?

*Unilateral facial paralysis involving the forehead is a common presentation in the ED with Bell’s palsy at the top of the differential. The work-up is often minimal.*

What makes this presentation of disease reportable?

*Unilateral facial paralysis involving the forehead due to bilateral necrotizing mastoiditis is an uncommon and life-threatening cause of this condition.*

What is the major learning point?

*To avoid missing more insidious pathology, clinicians should develop a robust differential when presented with unilateral facial paralysis involving the forehead.*

How might this improve emergency medicine practice?

*Maintaining a robust differential for unilateral facial weakness with forehead involvement will reduce misdiagnoses and delays in diagnosing serious mimics.*

## INTRODUCTION

Bell’s palsy is a sudden-onset, unilateral facial weakness primarily affecting the peripheral portion of the seventh cranial nerve.^1^ This results in a one-sided facial droop involving the forehead and palpebral fissures. It is the most common cause of unilateral facial weakness (60–70%) with an incidence of approximately 20 per 100,000 worldwide.^1^ The etiology is often unknown with most cases classified as idiopathic.^1^ ^–^ ^3^ Approximately 71% of cases resolve spontaneously, and 84% of patients regain near full function including facial muscle and nerve control.^1^ ^,^ ^4^ Due to this lack of a common etiology and the frequency of spontaneous improvement, a standardized treatment protocol for Bell’s palsy does not exist other than a possible short course of corticosteroids or antivirals.^1^ Given the characteristic physical exam findings in Bell’s palsy and the ability to differentiate from stroke using history and exam, most guidelines recommend against routine imaging in acute cases due to cost and lack of impact on the course of therapy.^5^

There is a subset of patients who have Bell’s palsy due to a more dangerous underlying pathology. Phenothiazine has been observed to cause a dystonic reaction that presents similarly to Bell’s palsy.^6^ Adenoid cystic carcinomas of the parotid gland can also be disguised as Bell’s palsy for years.^7^ These diagnoses may be missed due to the assumption that they fall in the same category as the vast majority of benign Bell’s palsy cases. We report a case of an 84-year-old female who presented to the emergency department (ED) with a left-sided facial droop that was consistent with Bell’s palsy, but was caused by bilateral bacterial necrotizing mastoiditis.

## CASE REPORT

An 84-year-old female with a past medical history of chronic leukopenia, chronic headaches, vertigo, hypertension, and osteoarthritis presented to the ED as a stroke alert from her outpatient otolaryngology appointment for progressive hearing loss and bilateral ear pain. Upon arrival, the patient revealed a four-day history of left-sided facial droop and a four-week history of headaches, describing the pain as originating from both ears radiating to her jaw. The patient also noted bilateral tinnitus over the prior 2–3 weeks with decreased hearing and ear swelling. She denied any significant change in the chronic periodic vertigo, which she had suffered for years. She also denied any peripheral numbness, tingling, or weakness.

Physical examination found a well-appearing elderly female in no acute distress. Vital signs were concerning for hypertension (184/89 millimeters of mercury) but were otherwise within normal limits. The patient had an obvious left-sided facial droop, with paralysis of the left-sided facial muscles involving the forehead and palpebral fissures. She was unable to raise her left eyebrow but was able to close her eyes bilaterally. Pupils were equal and reactive to light with intact extraocular motions. The remainder of the neurologic examination was reassuring with normal mental status, no strength or sensory deficits in bilateral upper and lower extremities, normal finger-to-nose test, no visual field deficits, and an ability to ambulate with a steady gait. There was significant swelling and tenderness of the external auditory canals and mastoids bilaterally. The tympanic membranes were non-visualized bilaterally due to significant canal edema. All other physical exam findings were unremarkable.

**Image 1.**
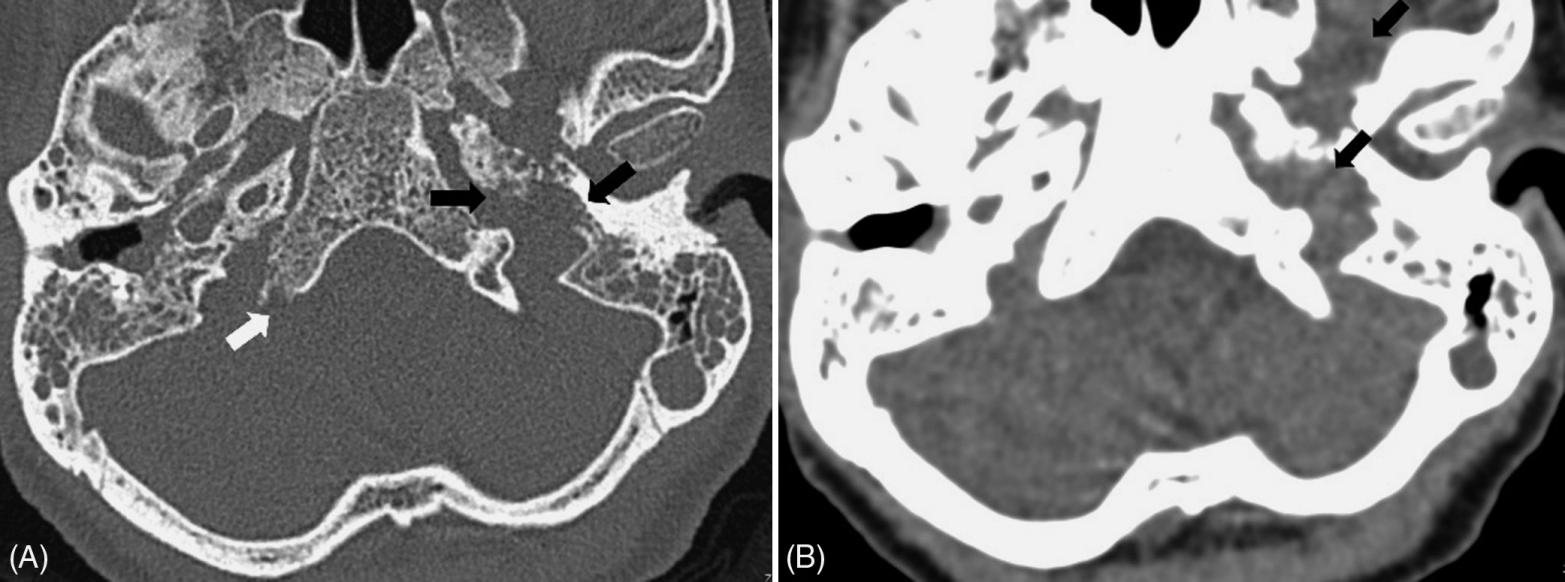
Axial computed tomography bone (A) and soft tissue window (B) images demonstrate opacification of the bilateral mastoid air cells and right middle ear cavity, compatible with otomastoiditis. Image A shows erosive changes in the right posterior clivus (white arrow) and left petrous apex and jugular foramen (black arrows). Image B shows abnormal soft tissue seen in the left jugular foramen and masticator space (black arrows).

The patient’s notable mastoid process tenderness, acute hearing loss, and inflammation of her auricles raised concern for an erosive or infectious process leading to her Bell’s palsy. Her facial paralysis involved the ipsilateral forehead, making stroke a less likely cause of her presentation. She had no other signs or symptoms on exam concerning for an acute embolic or hemorrhagic stroke including weakness or numbness in her limbs, ataxia, gait instability, or visual deficits. Due to the high suspicion for mastoiditis or other underlying infectious etiologies, a computed tomography (CT) brain and temporal bone was performed and revealed erosion of the mastoid segments of the greater facial nerve canals on the left side. The CT also demonstrated opacification of the mastoid air cells bilaterally with erosion of the bilateral proximal styloid processes (Image 1). New erosive and destructive changes were also seen in the right occipital condyle and right posterior clivus suspicious for an infectious or neoplastic process.

**Image 2.**
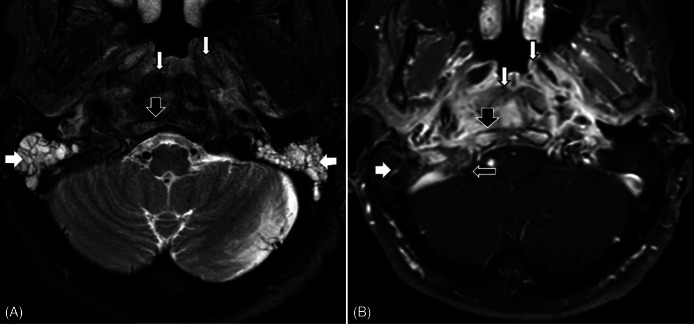
Axial T2 fat-suppressed image (A) and T1 fat-suppressed post-contrast image (B) through the nasopharynx, skull base, and mastoid air cells demonstrate opacification of the mastoid air cells bilaterally with enhancement of the septa on the right (wide white arrows) compatible with otomastoiditis. Edema and enhancement in the clivus (wide black arrows) compatible with skull base osteomyelitis. Edema, enhancement, and soft tissue thickening of the nasopharynx and prevetebral muscles (thin white arrows) compatible with extensive inflammation. Filling defect in the right sigmoid sinus and jugular bulb compatible with dural venous sinus thrombosis (thin black arrow).

Given these imaging findings, the patient was empirically treated with intravenous (IV) vancomycin and clindamycin for presumed erosive mastoiditis. Subsequent head and neck magnetic resonance imaging demonstrated widespread skull base osteomyelitis (SBO). There was noted to be involvement of the stylomastoid foramen bilaterally, extending along the left greater than right facial nerves, likely an extension of the infectious process. There was also likely sigmoid sinus thrombosis on the right extending into the jugular vein (Image 2).

Otolaryngology was consulted and placed bilateral ear wicks in the ED for topical ofloxacin administration. The next day, the patient underwent a bilateral myringotomy tube placement, adenoidectomy, and complete bilateral mastoidectomy. She was continued on IV antibiotics with transition to cefepime, vancomycin, and metronidazole for broader coverage while middle ear cultures taken in the operating room were pending. On post-operative day one, the patient had significant pain relief and improvement in her facial nerve function. Cultures from the mucoid discharge of the left middle ear grew *Pseudomonas aeruginosa*, and her antibiotic regimen was changed to oral ciprofloxacin based on culture sensitivities.

During her hospitalization a CT venogram also confirmed a right venous sinus thrombus, and the patient was placed on enoxaparin 80 milligrams four times daily for the next three months. She was discharged with a six-week course of oral ciprofloxacin and outpatient follow-ups with otolaryngology, neurology, infectious disease, and her primary care physician. The patient was seen in clinic three months later and was doing well with resolved facial nerve palsy, resolved headaches, and significantly improved hearing.

## DISCUSSION

This case represents a unique presentation of Bell’s palsy due to erosive mastoiditis and SBO. The patient presented with bilateral ear pain radiating down to her jaw with associated swelling and tenderness of the bilateral external auditory canals. These concerning symptoms, in addition to her several weeks of bilateral hearing loss, placed an erosive otogenic process high on the differential. As a result, it was decided to defer corticosteroids and pursue imaging. Although the medical team suspected an erosive process, SBO was an unexpected diagnosis.

Skull base osteomyelitis is a rare complication of infectious otogenic processes such as necrotizing mastoiditis. The soft tissue infection spreads from the mastoid process to the base of the skull via fissures and sutures, with resulting facial nerve palsy in up to 60% of cases.^8^ ^,^ ^9^ The most common causative organism in SBO is *P aeruginosa* (75–95%). Although SBO predominantly afflicts patients with uncontrolled diabetes,^9^ the patient presented in this case report did not have diabetes. Additionally, the clinical presentation is typically delayed and has a mortality of up to 20%.^9^ The patient in this case was incredibly well appearing and had no obvious risk factors apart from age and chronic leukopenia. This unique case raises the importance of maintaining a broad differential when approaching often benign pathologies such as Bell’s palsy, which are encountered in the ED.

When building this differential, it is important to consider recent research elucidating the multiple etiologies possibly responsible for Bell’s palsy.^10^ The leading cause currently is herpes simplex virus type 1 (HSV-1). This also correlates with the increased incidence of the palsy as patients age, as seroconversion of HSV-1 follows the same trend.^1^ ^,^ ^2^ Other causative viruses include varicella zoster virus, cytomegalovirus, and Epstein-Barr virus.^11^ A subset of Bell’s palsy is caused by bacterial pathogens, often following otitis externa, otitis media, or mastoiditis.^12^ A sequencing study performed in 2020 found that clinical samples from Bell’s palsy patients undergoing surgical decompression grew a variety of organisms. Viral, fungal, and bacterial organisms were all found in various patients’ samples with human betaherpesvirus 7, *Malassezia restricta*, and *P aeruginosa* being the most common etiologies, respectively.^11^ With the increasing number of causative agents being reported—some associated with serious sequelae—it is important for emergency physicians to apply due diligence when working up Bell’s palsy.

The literature shows multiple cases where a diagnosis of idiopathic Bell’s palsy was incorrectly made. In a retrospective cohort study of 43,979 patients across California EDs, 358 patients presenting with a unilateral facial paralysis were misdiagnosed with an idiopathic Bell’s palsy. The patients would be later diagnosed as having Guillain-Barré syndrome (10.9%), herpes zoster (23.2%), otitis media or mastoiditis (24.0%), or ischemic stroke (27%).^13^ Multiple cases show that involvement of the forehead does not rule out stroke entirely. Strokes within the dorsal pons have been reported in the literature to cause ischemia in the motor facial nerve and result in isolated unilateral facial paralysis involving the forehead.^13^

The effects of these misdiagnoses can be devastating, with some patients experiencing delays in care of up to three years for parotid malignancies due to their slow-onset facial paresis similar to Bell’s palsy.^14^ This data further emphasizes the importance of maintaining a broad differential for unilateral facial paralysis to prevent misdiagnoses and the resulting delays in care that will ultimately harm patients.

To reduce additional iatrogenic harm to these patients, it is critical to consider these less common etiologies prior to administering corticosteroids. Despite their effectiveness in alleviating symptoms in many Bell’s palsy patients, corticosteroids can also leave them vulnerable to underlying infection due to recruitment of T helper type 2 cells over type 1.^1^ ^,^ ^15^ In a case-control retrospective study of 2,632 critically ill patients exposed to corticosteroids, it was found that 46% developed secondary infections in the hospital with an increased incidence in mortality compared to 23% of the control group. If the patient in the currently reported case had been given corticosteroids without further evaluation, she could have suffered a significant worsening of her necrotizing mastoiditis. Therefore, it is important to evaluate these patients thoroughly for an underlying bacterial etiology of their facial palsy before initiating corticosteroids.

## CONCLUSION

Bell’s palsy is a common and often benign condition. The patient presented here had a presentation of Bell’s palsy in the setting of serious bilateral necrotizing mastoiditis complicated by skull base osteomyelitis. This underlying process was only caught due to thorough evaluation and special attention to red flags present in her history and physical examination. This case emphasizes the importance of maintaining a broad differential when approaching commonly benign pathologies such as Bell’s palsy. Maintaining a high clinical suspicion for the rarer etiologies of unilateral facial paralysis despite initial impressions will lead to more accurate diagnoses and fewer delays in care for those who need it most.
